# Body Weight Estimation for Pigs Based on 3D Hybrid Filter and Convolutional Neural Network

**DOI:** 10.3390/s23187730

**Published:** 2023-09-07

**Authors:** Zihao Liu, Jingyi Hua, Hongxiang Xue, Haonan Tian, Yang Chen, Haowei Liu

**Affiliations:** 1 College of Engineering, Nanjing Agricultural University, Nanjing 210031, China; 2 Key Laboratory of Breeding Equipment, Ministry of Agriculture and Rural Affairs, Nanjing 210031, China; 3 College of Artificial Intelligence, Nanjing Agricultural University, Nanjing 210031, China

**Keywords:** pig weight estimation, 3D sensor, point cloud segmentation, convolutional neural network

## Abstract

The measurement of pig weight holds significant importance for producers as it plays a crucial role in managing pig growth, health, and marketing, thereby facilitating informed decisions regarding scientific feeding practices. On one hand, the conventional manual weighing approach is characterized by inefficiency and time consumption. On the other hand, it has the potential to induce heightened stress levels in pigs. This research introduces a hybrid 3D point cloud denoising approach for precise pig weight estimation. By integrating statistical filtering and DBSCAN clustering techniques, we mitigate weight estimation bias and overcome limitations in feature extraction. The convex hull technique refines the dataset to the pig’s back, while voxel down-sampling enhances real-time efficiency. Our model integrates pig back parameters with a convolutional neural network (CNN) for accurate weight estimation. Experimental analysis indicates that the mean absolute error (MAE), mean absolute percent error (MAPE), and root mean square error (RMSE) of the weight estimation model proposed in this research are 12.45 kg, 5.36%, and 12.91 kg, respectively. In contrast to the currently available weight estimation methods based on 2D and 3D techniques, the suggested approach offers the advantages of simplified equipment configuration and reduced data processing complexity. These benefits are achieved without compromising the accuracy of weight estimation. Consequently, the proposed method presents an effective monitoring solution for precise pig feeding management, leading to reduced human resource losses and improved welfare in pig breeding.

## 1. Introduction

Pork is an essential source of animal protein and fat for European, Chinese, and some other populations, which plays a crucial role in ensuring food security and social stability [1,2,3]. In China, the pig industry is a traditionally advantageous industry that contributes greatly to sustaining the agricultural market, fostering the growth of the national economy, and increasing farmers’ incomes. As such, the quantity of pig breeding has witnessed a notable increase. However, conventional pig breeding methods have proven inadequate for keeping pace with the rapid development of pig output. The integration of science and technology has facilitated the widespread adoption of contemporary technologies in various aspects of aquaculture production.

Consequently, the improvement of pig production and quality has consistently remained a central objective in the development of agricultural strategies [4,5,6]. Among these objectives, pig weight stands out as one of the most influential factors in pig production and pork quality. It provides essential information on feed conversion rate, growth rate, disease, development uniformity, and the health status of pig herds [7,8,9,10]. Presently, the traditional manual pig driving weighing method involves direct contact weighing on a scale. This method is known for its time-consuming, labor-intensive nature, and is prone to causing the stress reaction in pigs. These limitations make it challenging to meet the requirements of large-scale breeding for real-time monitoring of pig weight [11].

**Figure 1:**
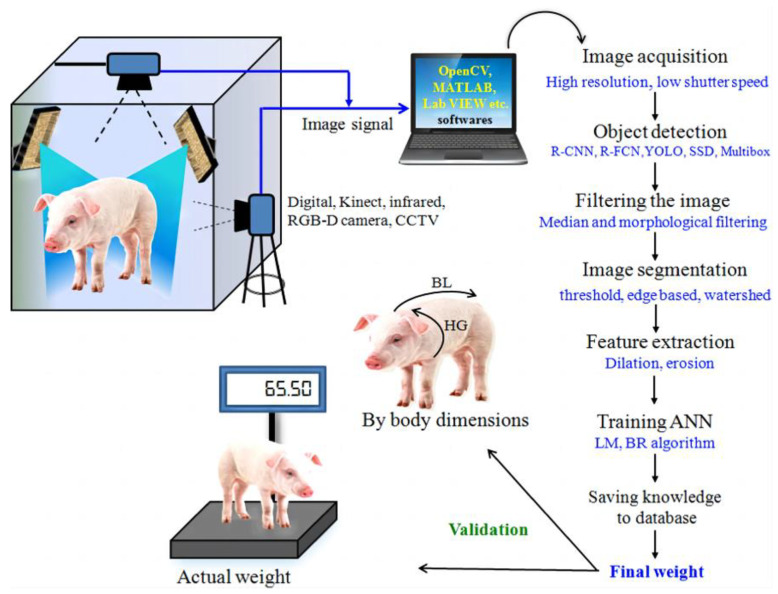
The fundamental stages involved in 2D image processing for the purpose of estimating pig weight [12].

Non-contact monitoring of pig weight has progressively become a hotspot for research, with weight estimation primarily relying on 2D and 3D images [12,13,14,15]. In 2D imaging, segmentation methods are employed to delineate the pig’s outline, while weight estimation is based on a relationship model using geometric parameters and actual body weight, as illustrated in Figure 1. In 2017, Okinda [16] used HD cameras to capture overhead images of pigs, applied the background difference method to eliminate background interference, and devised a weight estimation model using linear regression and adaptive fuzzy reasoning. This model necessitates manual selection of optimal inference images due to the influence of porcine posture and movement. In 2018, Suwannakhun [17] et al. used an adaptive threshold segmentation method to identify the pig’s body region and developed a body weight estimation model leveraging eight features, including color, texture, center of mass, main axis length, minimum axis length, eccentricity, and area, to mitigate the impact of pig movement. Nevertheless, 2D methods for estimation often fall short in accurately estimating the animal’s volume or parameters associated with its body surface. Furthermore, variations in camera parameters, object distance, and lighting conditions have a significant impact on the measurement findings, resulting in limited adaptability and posing challenges in enhancing accuracy in real-world multi-scene applications. In the context of weight regression estimation, the conventional regression approach exhibits limited capability in feature learning and fails to comprehensively capture the interplay among the defining factors of pig body size.

**Figure 2:**
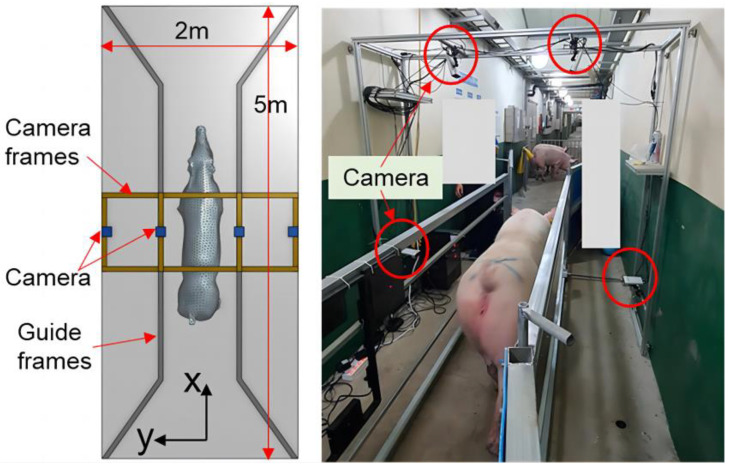
Configuration of the acquisition system for point clouds [20].

Research aimed at estimating the weight of pig based on three-dimensional data has been undertaken to address these problems. Liu [18] et al. used binocular vision technology to collect three-dimensional image data of pigs. They extracted growth-related data, including chest circumference, buttock height, body length, body height, and body width, through image processing methods. After that, they developed the estimation model based on linear functions, nonlinear functions, machine learning algorithms, deep learning algorithms, among others, to estimate pig weight. The correlation coefficient with the measured value was 97%, and the average relative error was 2.5%, outperforming models solely relying on back area measurements. As the price of depth cameras continues to fall, and their performance steadily improves, they have been increasingly employed in studies of animal body measurement, body condition and weight estimation, automatic behavioral identification, etc. In 2021, He [19] et al. used a D430 camera to capture depth images of pigs. They employed instance segmentation, distance independence, noise reduction, and rotational correction to eliminate background interference and constructed a BotNet-based model for estimating the body weight of pigs. In 2023, Kwon [20] et al. introduced a method for accurately estimating pig weight using point clouds, as depicted in Figure 2. They used a rapid and robust reconstructed grid model to address the problem of unrecognized noise or missing points and optimized deep neural networks for precise weight estimation. While the aforementioned techniques have demonstrated enhanced precision in estimating the weight of pigs, it is crucial to acknowledge that the hardware systems employed in these experiments are intricate. In real-world production settings, the available space is often limited, hindering the ability to conduct multi-directional or azimuthal livestock detection. Meanwhile, data collection conditions may not be optimal, posing challenges in meeting current manufacturing demands.

In conclusion, there is a pressing requirement for a precise and cost-effective intelligent system to estimate pig weight accurately. Such a system should be applicable to large-scale pig farms, facilitating the monitoring and analysis of pig growth processes and serving as an effective tool for precise pig feeding and management.

The primary contributions of this article can be summarized as follows:

(1) Point cloud filtering, the denoising of large-scale and small-scale discrete points is achieved using statistical filtering and DBSCAN point cloud segmentation, respectively, resulting in the accurate segmentation of the pig back image. What is more, the voxel downsampling approach is employed to reduce the point cloud data’s resolution on the pig’s back, so as to reduce the volume of point cloud data while preserving the inherent characteristics of the original dataset.
(2) In terms of the weight estimation, the plane projection technique is employed to determine the body size of the pig, while the convex hull detection method is utilized to eliminate the head and tail regions of the pig. The point set information in the pig point cloud data is input into the convolutional neural network to construct the pig weight estimation model, which realizes the full learning of features and accurate estimation of pig weight.
(3) As for actual breeding, the estimation method constructed in this research overcomes the problems of high cost, data processing difficulties, space occupation, and layout restrictions while ensuring the estimation accuracy, which can provide technical support for precise feeding, selection, and breeding of pigs.

This paper is structured as follows: Section 2 introduces the point cloud data collection, noise reduction segmentation, feature extraction, and weight estimation methods; in Section 3, the performance of denoising segmentation and the weight estimation model is analyzed. Section 4 discusses the comparison with existing methods, the limitations, improvements of the research, and outlines potential future work. Finally, Section 5 presents the conclusions.

## 2. Materials and Methods

**Figure 3:**
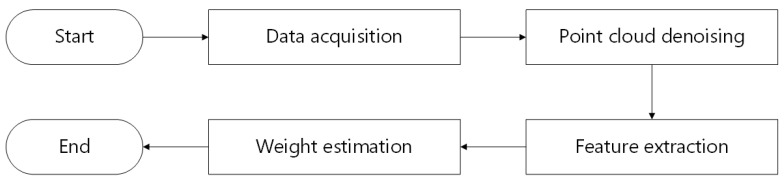
The technical path.

This section primarily describes the experimental objects and techniques employed. Figure 3 depicts the technical path of this research.

### 2.1. Animals and Housing

The experimental period spanned from 7 June to 27 June 2022 and the experimental site was located in Wens Pig Farm, Xinxing County, Yunfu City, Guangdong Province, China. The experimental subjects were 198 pigs (Large White × Landrace breed), with body weights ranging from 190 kg to 300 kg. The equipment was installed in the passageway connecting the gestation house to the farrowing house of the farm, which had a width of 1.7 m and a height of 2.8 m. All experimental design and procedures conducted in this study received approval from the Animal Care and Use Committee of Nanjing Agricultural University, in compliance with the Regulations for the Administration of Affairs Concerning Experimental Animals of China (Certification NJAU.No20220530088).

### 2.2. Data Acquisition

**Figure 4:**
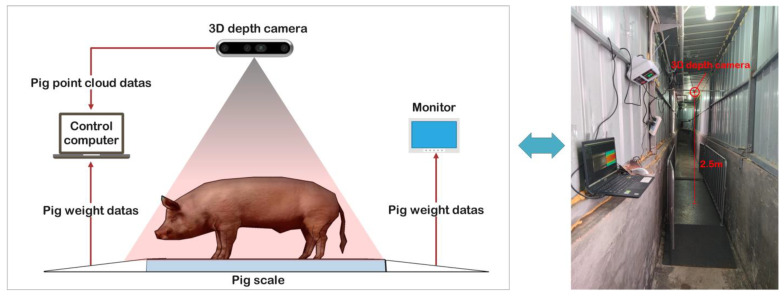
The pig weight data acquisition system.

A 3D depth Intel RealSense D435i camera (manufactured by Intel Corp., Santa Clara, CA, USA) was used to acquire color and depth images. This camera features a complete depth processing module, a vision processing module, and a signal processing module, capable of delivering depth data and color information data at a rate of 90 frames per second. What is more, it was installed at 2.5 m directly above the XK3190-A12 weighing scale (manufactured by Henan Nanshang Husbandry Technology Co., Ltd., Nanyang, China). An illustration of the pig weight data acquisition system is presented in Figure 4.

### 2.3. Data Set Construction

**Figure 5:**
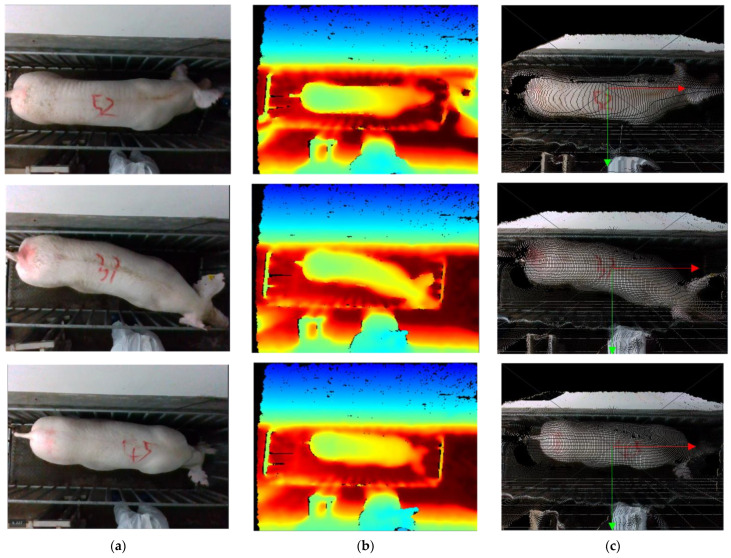
The dataset fragment. (**a**) Color images; (**b**) Depth images; (**c**) color images after mapping.

To improve the efficiency of data acquisition, this research used ROS parsing for video parsing to acquire 3D point cloud data in PLY format. Each data set comprises a color image and a depth image, captured at a resolution of 640 × 480 pixels and a frame rate of 30 frames per second. A portion of the dataset fragment is depicted in Figure 5.

**Figure 6:**
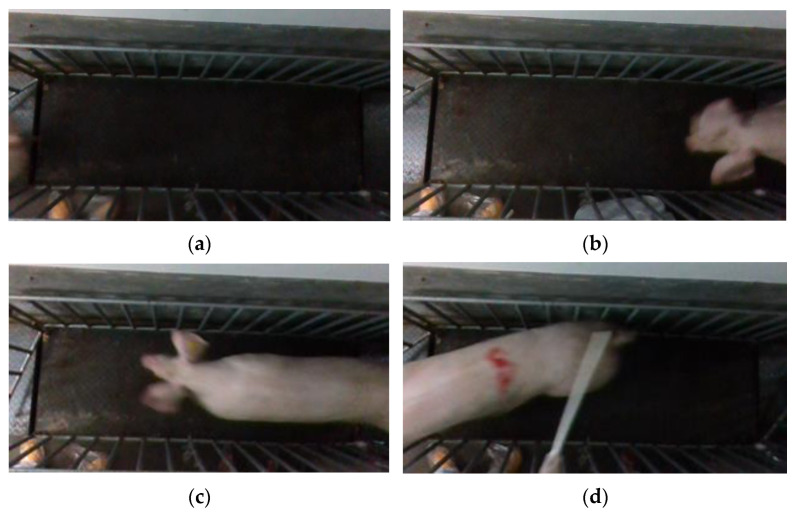
Incomplete pig picture. (**a**) No pig; (**b**) pig with missing body; (**c**) pig with missing rump; (**d**) pig with missing head.

Point cloud files with missing point clouds and incomplete pig images were excluded, as shown in Figure 6. Only point cloud files featuring complete pig images were retained. To ensure data sufficiency and meet the requirements of the pig weight estimation model, 50 point cloud files were selected for each pig. Finally, a total of 10,000 sets of pig 3D point cloud data were acquired.

### 2.4. Model Construction

#### 2.4.1. Overall Process

**Figure 7:**
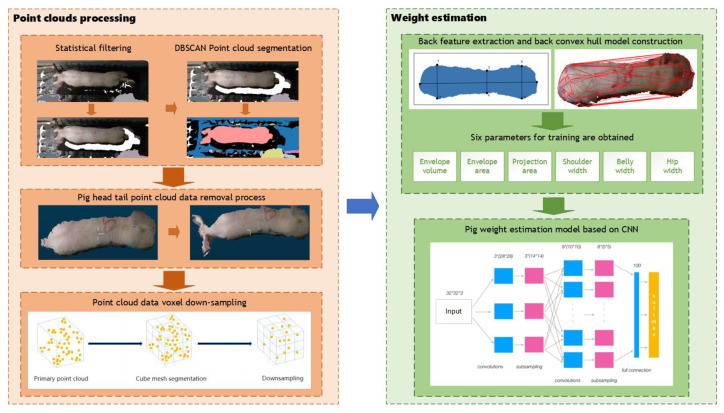
Overall process.

The primary components of the method proposed in this research are point cloud segmentation and weight estimation, as illustrated in Figure 7. In order to address the adhesion problem between pigs and the background in the segmentation of pig point cloud, statistical filtering and DBSCAN was used to separate pig point cloud from background. To obtain complete point cloud data of the pigs’ backs, the plane projection is employed to capture images of the pig’s back. After that, convex hull detection was applied to remove extraneous head and tail point cloud data. To reduce the computational complexity of subsequent processing, voxel down-sampling was used to reduce the quantity of point cloud data on pigs’ backs. Finally, for precise pig weight estimation, we devised a weight estimation model based on back parameter characteristics and a convolutional neural network (CNN) during the weight estimation phase.

#### 2.4.2. Data Denoising

The collected data contain some three-dimensional point cloud noise, attributed to occluders, equipment limitations, and external conditions [21]. This noise can compromise the accuracy of expressing useful information, which results in a large error in pig weight estimation. To address this issue, the noise points within the point cloud were separated into large-scale outlier points and small-scale discrete points based on their distribution characteristics. Prior research has demonstrated that employing a diverse range of hybrid filtering algorithms tailored to specific attributes of noise might yield superior outcomes [22]. In this research, a statistical filter was used to remove large-scale discrete points, and DBSCAN was used to remove small-scale discrete points.

Preserving the local features of point cloud data is of utmost importance when capturing the physical characteristics of pigs. Within the collected 3D point cloud data, there exists large-scale noise, primarily consisting of sparse points that deviate from the pig’s body point cloud and hover above it. What is more, there are smaller, denser point clouds that are more distant from the center of the pig’s body point cloud. In the present, for the removal of such discrete points, techniques like passthrough filtering, statistical filtering, conditional filtering, guided filtering, and others are usually employed [23]. Passthrough filtering serves as an effective manner in removing noise in large-scale backgrounds. What should be noticed is that it almost does not work when it comes to randomly generated discrete noise and may even lead to inadvertent removal of pig point cloud data.

This study mainly employs the open-source library Open3D (version 0.13.0) to process three-dimensional point cloud images. Open3D is an open-source library that is available in the rapid development of software for handling 3D data. The statistical filtering algorithm employs the distance between the queried point in the point cloud data and all neighboring point sets in its vicinity to perform statistical analysis and eliminate large-scale discrete points [24]. When the point cloud density of a particular location falls below the predetermined threshold, these points are removed as discrete points. The exact procedure is outlined as follows:

Read into the 3D point cloud data set $P(p_{1},p_{2},...,p_{n})$, and establish the k-d tree data structure of the point cloud data.

For each point $p_{i}$ in the point cloud, define the required nearest neighbor parameter k, establish the $k_{nn}$ neighborhood based on the value of k, and calculate the average distance from the nearest k nearest neighbor, as shown in Equations (1) and (2), where $d_{ij}$ is the spatial distance between point $p_{i}$ and point $p_{j}$, and ${\bar{d}}_{i}$ is the average distance between point $p_{i}$ and its k nearest neighbors.

(1) $$d_{ij}=\sqrt{{\left(p_{ix}-p_{jx}\right)}^{2}+{\left(p_{iy}-p_{jy}\right)}^{2}+{\left(p_{iz}-p_{jz}\right)}^{2}}$$

(2) $${\bar{d}}_{i}=\frac{1}{k}\sum_{i=1}^{j=k}\;d_{ij}$$

On the basis of the last step, calculate the average distance ${\bar{d}}_{ni}$ of the average distance ${\bar{d}}_{i}$ of all k neighbors and the standard deviation $d_{std}$, as shown in Equation (3):

(3) $$d_{std}=\sqrt{\frac{\sum_{i=1}^{n}\;{\left({\bar{d}}_{ni}-{\bar{d}}_{i}\right)}^{2}}{n-1}}$$

Calculate whether the average distance ${\bar{d}}_{i}$ between the current $p_{i}$ and the k nearest neighbor is greater than the set threshold *L*. When ${\bar{d}}_{i}$ > *L*, delete point $p_{i}$; when ${\bar{d}}_{i}$ < *L*, retain point $p_{i}$. As shown in Equation (4), σ is the calculated coefficient, which is generally valued according to the distribution of the cloud data of the measured point.

(4) $$L={\bar{d}}_{ni}+\sigma \times d_{std}$$

**Figure 8:**
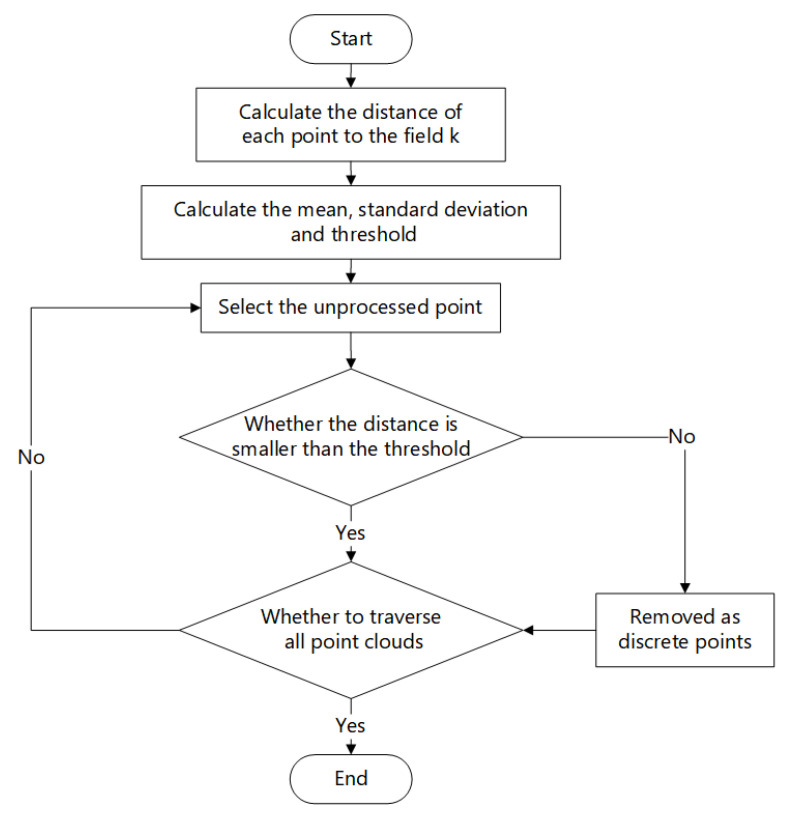
Statistical filtering process diagram.

Traverse all points in the point cloud for calculation until all point cloud data is processed and filtered point cloud data is output. The statistical filtering process is presented in Figure 8.

Using statistical filtering for denoising 3D point cloud data can remove some of the large-scale noise, though there still exists some. These noises mixed with the pig’s data point can interfere with the surface smoothness of 3D model reconstruction. To address this issue, a secondary denoising step is required, incorporating additional filtering algorithms. Employing a hybrid filtering approach serves a dual purpose—it removes small-scale noise and mitigates the remaining large-scale noise. Clustering algorithms help tackle small-scale noise, to certain degree. By identifying points with high similarity within clusters, noise points can be distinguished from valid data points. Clustering algorithms usually include K-Means clustering, DBSCAN clustering, region growing clustering, etc. K-Means clustering relies on geometric distance and proves advantageous when dealing with point clouds where the number and location of seed points are known [23]. However, when applied to interconnected point clouds, it may cause issues such as over-segmentation or under-segmentation due to the reliance on single growth criteria or stopping conditions. In contrast, DBSCAN clustering leverages point cloud density for clustering. During the clustering process, it randomly selects a core object as a seed and proceeds to identify all sample sets that are density-reachable from this core object. This process repeats until all core objects are clustered. DBSCAN clustering excels in situations where different regions of the pig’s body exhibit similar point cloud densities, coming up with robust clustering results.

**Figure 9:**
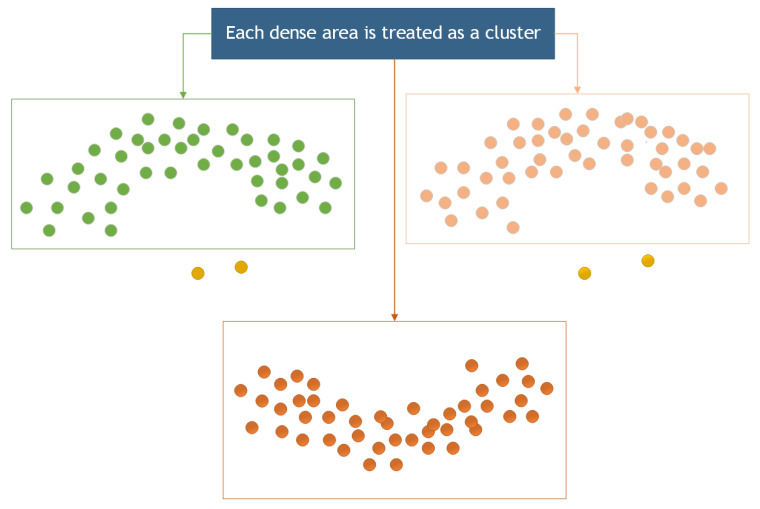
Cluster region division.

The density clustering algorithm has been employed to remove noise from a point cloud because of its sensitivity and effectiveness in recognizing small-scale noise points [25]. In this section, the Density-Based Spatial Clustering of Applications with Noise (DBSCAN) algorithm was for noise removal from the point cloud. DBSCAN is an algorithm for density-based spatial clustering that clusters regions with sufficient density [26]. It also defines a cluster as the largest set of density-connected points, allowing for the identification of clusters with arbitrary shapes in a noisy spatial database [27,28], as illustrated in Figure 9.

**Figure 10:**
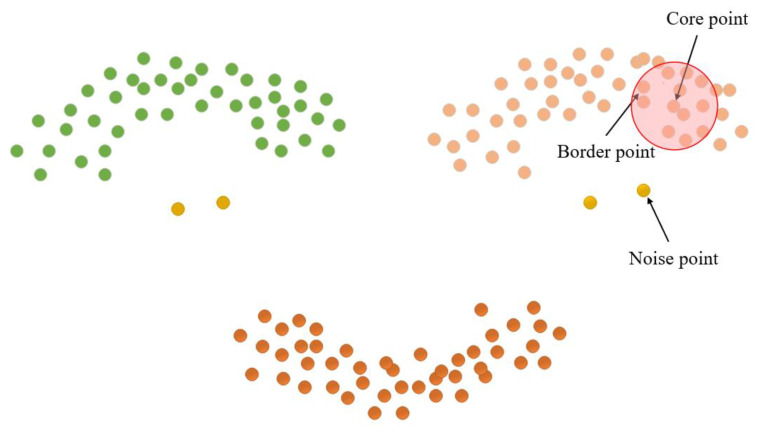
Cluster point classification.

**Figure 11:**
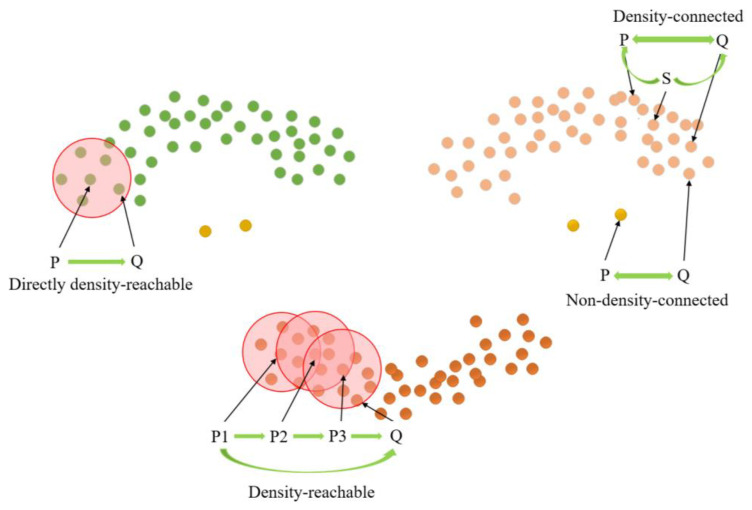
Cluster density relation.

The length parameter epsilon (Eps) and minimum points (MinPts) are crucial factors in determining the clustering effect of the DBSCAN clustering algorithm [29]. MinPts sets the threshold for the minimum number of data points needed to form a density region, while Eps quantifies the distance between a data point and its neighbors. Core points, border points, and noise points can be derived from these two parameters, as illustrated in Figure 10. Notably, there exist four relationships between these three data points: Directly density-reachable, density-reachable, density-connected, and non-density-connected, as illustrated in Figure 11.

The pseudocode for the clustering algorithm based on DBSCAN used in this research is shown in Algorithm 1.

  **Algorithm 1.** The pseudocode for the clustering algorithm based on DBSCAN  **Input:** Datasets *Ds*, parameter *c* and *MinPts*

  **Output:** Clustering result Cl

  DBSCAN (*Ds*, *c*, *MinPts*)

  *Cl* ← 0  every point in *Ds* is unlabeled

**  for** each unlabeled point *p* ∈ *Ds* do

    mark *p* as labeled

    set c_neighborhood as *p*’s c_neighborhood

    **if** sizeof (c_neighborhood) < *MinPts* then

      mark *p* as noise point

    **else**

      *Cl* ← *Cl* + 1

      add *p* to *Cl*

      neighbor ← c_neighborhood

      **for** each point *q* in neighbor

        **if**
*q* is unlabeled

          mark *q* as labeled

          set c_neighborhood’ as *q*’s c_neighborhood

          **if** sizeof (c_neighborhood’) >= *MinPts* then

            neighbor ← neighbor ∪ c_neighborhood’

          **end if**

        **if**
*q* is not assigned to any cluster

          add *q* to cluster *Cl*

        **end if**      **end for**

    **end if**
  **end for**


#### 2.4.3. Splitting the Head and Tail

**Figure 12:**
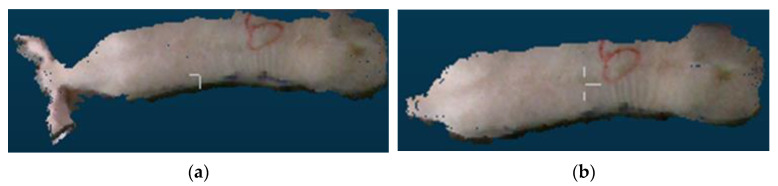
Comparison before and after head and tail removal: (**a**) original image; (**b**) processed image.

It is necessary to remove the head and tail point cloud data because the poses of pigs’ heads and tails frequently change, which has a remarkable impact on such essential characteristic parameters as the envelope volume, envelope surface area, back area, and body length. Initially, the data from the point cloud were projected onto a plane to create a 2D outline of the pig’s back. The minimum envelope polygon of the pig’s body was derived from the envelope vertices and line segments corresponding to the pig’s head and tail. The sag degree of the pig contour was calculated based on the point where the contour envelope overlapped with the pig’s contour and the pixel distance between neighboring points and the pig’s contour. A deeper depression indicated a higher probability of head and tail split points. Given the fixed direction of the pig’s head, the short axis of some particles in the pig’s body was chosen as the dividing line, and the point furthest from this axis was considered the dividing point of the tail, while the point closest to the axis was considered the dividing point of the head. Figure 12 depicts the comparison before and after head and tail removal.

#### 2.4.4. Point Cloud Sampling

This research performs voxel down-sampling of point cloud data because the amount of 3D point cloud data is exceedingly large and irregular, which has a profound effect on the model’s input. The particular procedure is as follows [30]:

Based on the point cloud data within the dot cloud files, the maximum and minimum values of X, Y, Z in the 3D space coordinates are obtained. The maximum values of the 3D coordinates are represented as x_max_, y_max_, z_max_, while the minimum values are denoted as x_min_, y_min_, z_min_. We used NumPy libraries to construct a variation of the synchronized function, generating an n × 3 matrix concerning x, y, z. Each entry represents the 3D coordinates xyz of the point, with the relationships between z, x, and y as follows:

(5) $$z=\frac{\sin(x^{2}+y^{2})}{x^{2}+y^{2}}$$

The side length of the regular cube grid is represented as r. The smallest bounding body of the point cloud data is determined by the maximum and minimum values of 3D coordinate points in the point cloud data.

Calculate the size of the voxel grid.

(6) $$\left\{\begin{array}{c} D_{x}=\frac{\left(x_{max}-x_{min}\right)}{r}\\ D_{y}=\frac{\left(y_{max}-y_{min}\right)}{r}\\ D_{z}=\frac{\left(z_{max}-z_{min}\right)}{r} \end{array}\right.$$

Calculate the index of each regular small cube grid, and the index is represented by h as follows:

(7) $$\left\{\begin{array}{c} h_{x}=\frac{\left(x-x_{min}\right)}{r}\\ h_{y}=\frac{\left(y-y_{min}\right)}{r}\\ h_{z}=\frac{\left(z-z_{min}\right)}{r}\\ h=h_{x}+h_{y}\times D_{x}+h_{z}\times D_{x}\times D_{y} \end{array}\right.$$

Sort the elements in h by size, and then calculate the center of gravity for each regular cube to represent each point in the small grid.

#### 2.4.5. Back Feature Extraction

Several studies have demonstrated a correlation between pig body measurement, body weight, and other growth parameters. A relationship model can be used to estimate the body weight of pigs in an effective manner [31,32]. Body size parameters were extracted based on critical points along the back contour of the pigs, and a relationship model between body size and body weight was established.

**Figure 13:**
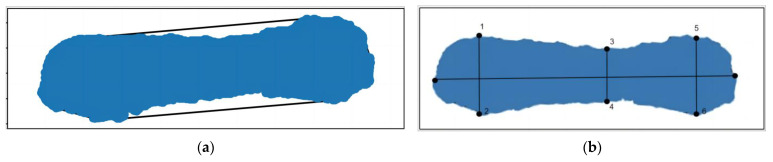
Pig point cloud projection. (**a**) Before horizontal processing; (**b**) after horizontal processing.

During the process of acquiring pig 3D point cloud data, the pig’s body is often inclined, making it difficult to extract body size parameters. In this research, a minimum horizontal bounding box was created for the point cloud on the pig’s back. The bounding box maintained the parallelism of the border with the X and Y axes, ensuring that the rear image remained horizontal. As shown in Figure 13, the 3D point cloud data were projected onto the horizontal plane to obtain the horizontal projection of the point cloud from the pig’s back.

The six key points along the pig’s outline were extracted. L2 represented the shortest distance between the two ends of the outline, while L1 represented the greatest distance between the ends of the left side and L3 signified the longest distance between the ends of the right side. The calculation formula is as follows, where *x* and *y* represent the coordinates of the respective points on the plane:

(8) $$L1=\sqrt{{\left(x_{1}-x_{2}\right)}^{2}+{\left(y_{1}-y_{2}\right)}^{2}}$$

(9) $$L2=\sqrt{{\left(x_{3}-x_{4}\right)}^{2}+{\left(y_{3}-y_{4}\right)}^{2}}$$

(10) $$L3=\sqrt{{\left(x_{5}-x_{6}\right)}^{2}+{\left(y_{5}-y_{6}\right)}^{2}}$$

**Figure 14:**
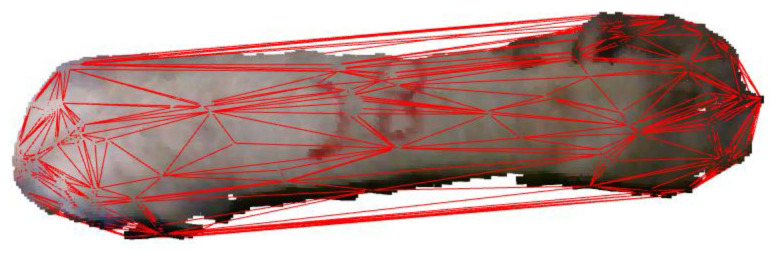
Pig back envelope.

Then, the convex hull model of the pig back was constructed, and the point cloud of the pig back was encapsulated in the form of minimum convex hull so that the area and volume of the convex hull model could be calculated. The extraction results are depicted in Figure 14.

#### 2.4.6. Weight Estimation

Nowadays, methods for estimating pigs’ weight mainly include three groups: classical regression methods (including Linear Regression, Polynomial Regression, Ridge Regression, and Logistic Regression), machine learning methods (including Decision Tree Regression, Random Forest Regression, and Support Vector Regression, SVR), and deep learning methods (like Multi-Layer Perceptron, MLP, and Convolutional Neural Network, CNN). Classical regression models, though commonly used, may be instable due to the nonlinear relationships and complex features among body feature parameters. Machine learning regression methods, though flexible in handling nonlinear relationships, can be sensitive to outliers and noise within the training data. In contrast, deep learning-based regression methods excel in handling nonlinear patterns and demonstrate great scalability compared to the first two groups of regression estimation methods. CNN possesses strong data-solving capabilities and can delve deeply into data relationship information [33]. As a network with multiple layers, CNN consists primarily of input layer, convolutional layer, down-sampling layer, fully connected layer, and output layer [34]. Its fundamental concept lies in the use of neuron weight sharing, which reduces the diversity of network parameters, simplifies the network, and enhances execution efficiency [35].

Input layer: The initial sample data are input into the network to facilitate the feature learning of the convolutional layer, and the input data are preprocessed to ensure that the data align more closely with the requirements of network training.

Convolution layer: In this layer, the input features from the previous layer undergo convolution with filters, and the biased top is added. Nonlinear functions are applied to obtain the final output of the convolutional layer.

The output of the convolution layer is expressed as follows:

(11) $$u_{j^{\prime }}^{l}=\sum_{i^{\prime }\in R_{i^{\prime }}}\chi_{j^{\prime }}^{l-1}*W_{i^{\prime }j^{\prime }}^{l}+B_{j^{\prime }}^{l}$$

(12) $$\chi_{j^{\prime }}^{l}=f\left(u_{j^{\prime }}^{l}\right)$$

where $\chi_{j^{\prime }}^{l-1}$ represents the activation value of feature $j^{\prime }$ in layer *l* − 1, $W_{i^{\prime }j^{\prime }}^{l}$ represents the convolution kernel of layer *l* feature $j^{\prime }$ and the previous layer feature $i^{\prime }$, while B denotes the bias value. $u_{j^{\prime }}^{l}$ describes the weighted sum of feature $u_{j^{\prime }}^{l}$ in the *l* layer, and $f\left(\cdot \right)$ represents the activation function, with ∗ indicating the convolution operation.

All outputs are connected with the adjacent neurons in the upper layer, which helps train the sample features, reduce the parameter connections, and improve learning efficiency.

Down-sampling layer: In this layer, the output features are processed via pooling using the down-sampling function. After that, weighted and biased calculations are performed on the processed features to obtain the output of the down-sampling layer:

(13) $$u_{j^{\prime }}^{l}=\beta_{j^{\prime }}^{l}down(\chi_{i^{\prime }}^{l-1})+B_{j^{\prime }}^{l}$$

(14) $$\chi_{j^{\prime }}^{l}=f\left(u_{j^{\prime }}^{l}\right)$$

where β represents the weight coefficient, and down(·) is the down-sampling function.

Fully connected layer: In the fully connected layer, all two-dimensional features are transformed into one-dimensional features, which serve as the output of the layer. And the output of the layer is obtained through weighted summation:

(15) $$\chi^{l}=f(u^{l})$$

(16) $$u^{l}=\omega^{\prime l}\chi^{l-1}+B^{l}$$

Output layer: The main job is to classify and identify the feature vector obtained by the CNN model and output the result.

The training process of CNN mainly involves two phases: the forward propagation phase and the backward propagation phase. During the former one, input data are processed through intermediate layers to generate output values and a loss function. In the latter phase, when there is an error between the output values and the target values, the parameters of each layer in the network are optimized through gradient descent based on the loss function.

### 2.5. Model Evaluation

To test the performance of the pig weight estimation model, metrics such as the mean absolute error (MAE), mean absolute percentage error (MAPE), and root mean square error (RMSE) were calculated by comparing the actual weight of the pig with the estimated weight.

(17) $$MAE=\frac{1}{N}\sum_{k=1}^{N}|P_{k}-R_{k}|$$

(18) $$MAPE=\frac{1}{N}\sum_{k=1}^{N}\frac{|P_{k}-R_{k}|}{R_{k}}\times 100\%$$

(19) $$RMSE=\sqrt{\frac{1}{N}\sum_{k=1}^{N}{\left(P_{k}-R_{k}\right)}^{2}}$$

where P represents the estimated value of the model, R represents the actual value, N signifies the number of samples, and k denotes the current sample number.

## 3. Results

### 3.1. Performance Comparison of Different Point Cloud Filtering Methods

**Figure 15:**
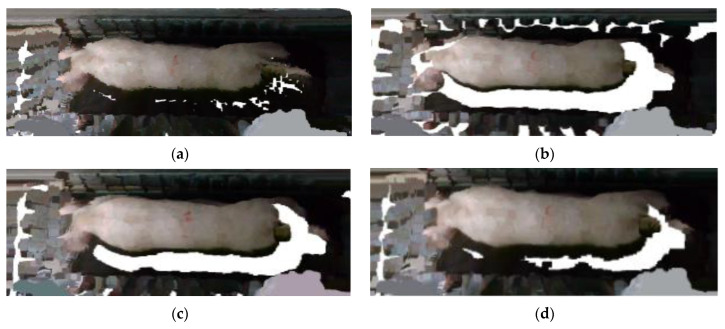
Comparison of statistical filtering effects: (**a**) Original image; (**b**) filtering effect with a threshold of 1; (**c**) filtering effect with a threshold of 2; (**d**) filtering effect with a threshold of 4.

To investigate the filtering effects of different thresholds, a comparative experiment was conducted with thresholds of 1, 2, and 4, and neighborhood points of 30. Figure 15 depicts the filtering effects of distinct thresholds. When the threshold value is set to 0.1, there is excessive filtering of the pig point cloud, which leads to partial loss of pig head point cloud data. On the other hand, when the threshold is set to 2, most of the discrete points can be effectively eliminated. When the threshold is set to 4, only a small number of discrete points are removed, and the impact is not apparent. Consequently, a threshold value of 2 was selected for the statistical filtering parameter in this study.

**Figure 16:**
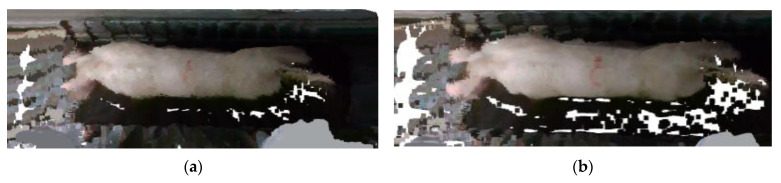
Comparison of guided filtering effect: (**a**) Original image; (**b**) guided filtering effect image.

To compare the filtering effects of different filters, the guided filter was used as a comparison in this research. The guided filter is a kind of edge-preserving smoothing method commonly employed in image enhancement, image dehazing, and other applications [36]. Figure 16 depicts the effect of guided filtering. The comparison reveals that while the guided filter was capable of preserving the entire point cloud data of the pigs, it did not filter the discrete point cloud data from the surrounding environment of the pigs. That is why the filtering effect was worse than that of statistical filtering.

### 3.2. Performance Comparison of Different Segmentation Methods for Point Cloud

**Figure 17:**
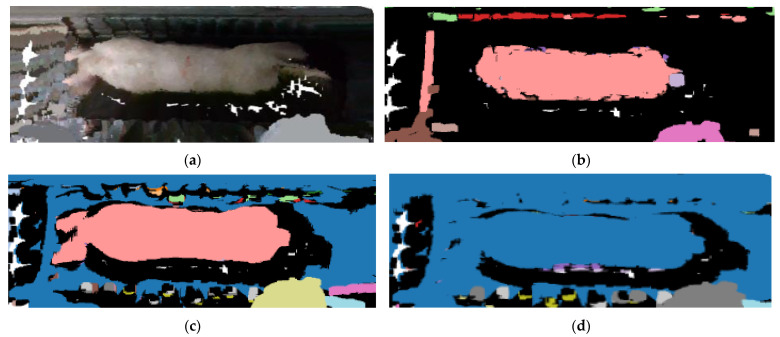
Comparison of DBSCAN Point cloud segmentation with different Eps value: (**a**) Original image; (**b**) segmentation effect with an Eps of 0.01; (**c**) segmentation effect with an Eps of 0.02; (**d**) segmentation effect with an Eps of 0.025.

For the purpose of exploring the influence of different radius of neighborhood on the clustering effect, a comparative experiment was conducted in this study using the radius of neighborhood (Eps) of 0.01, 0.02, and 0.025. The clustering effect of different Eps is shown in Figure 17. When Eps is set to 0.01, the pig’s body trunk is divided, but the head and tail are categorized as background. Also, the outline edge of the pig body is blurred, and the point cloud part of the pig is missing. Setting Eps to 0.02 bring about a complete separation of the pig, with a clear and smooth outline. When the Eps is set to 0.025, parts of the pig outline become visible, but the pig cannot be separated from the background. Therefore, the threshold value for Eps was set to 0.02 as the Eps of DBSCAN point cloud density clustering.

**Figure 18:**
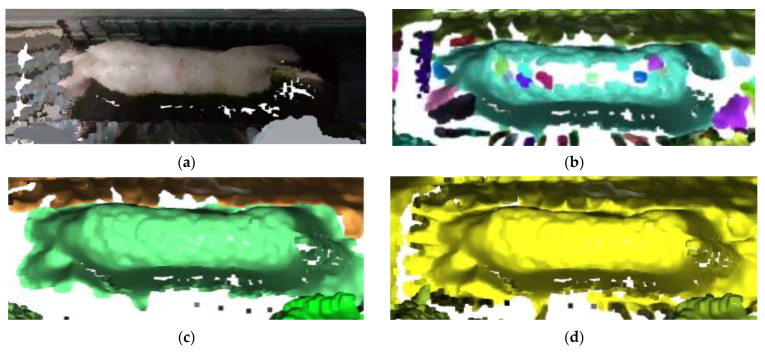
Comparison of segmentation effect of region growing segmentation for point cloud: (**a**) Original image; (**b**) segmentation effect with a ct of 0.01; (**c**) segmentation effect with a ct of 0.02; (**d**) segmentation effect with a ct of 0.05.

This research selected the region growing segmentation for point cloud as a reference for comparing the effects of various cloud segmentation methods. The region growing segmentation method is recognized for its flexibility, preservation of similarity, extensibility, computational efficiency, and interpretability, making it one of the most favored methods in point cloud segmentation [37]. The algorithm selects the seed point as the starting point and expands the seed region by adding neighboring points that meet specific conditions [38]. The curvature threshold (ct) is an important parameter influencing the region growing segmentation algorithm. Figure 18 illustrates the segmentation outcomes under different curvature thresholds. When ct = 0.01, a substantial portion of the pig’s back point cloud is missing, and some is erroneously classified as outliers. With ct = 0.02, the number of clusters in the point cloud data decreases, which brings about substantial filling of the pig’s back point cloud. Moreover, the segmentation correctly identifies and categorizes pig back point clouds as the primary class, but the issue of pig point cloud adhesion to the background remains severe. Finally, when ct = 0.05, the pig point cloud blends with the background, which means a failure. In comparison to the region growing segmentation algorithm, the DBSCAN-based segmentation model demonstrates greater robustness in handling parameters and noise. DBSCAN, as a clustering algorithm, offers advantages in point cloud segmentation tasks, especially in settings where there is no need to predefine the number of clusters. It exhibits robustness against noise and outliers while being capable of handling clusters with irregular shapes.

### 3.3. Analysis of Down-Sampling Results

**Figure 19:**
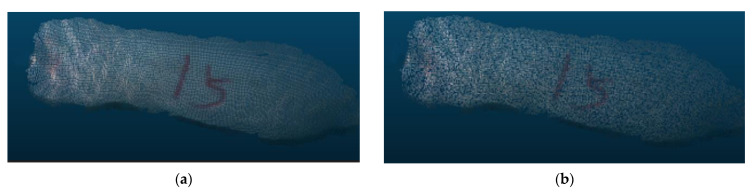

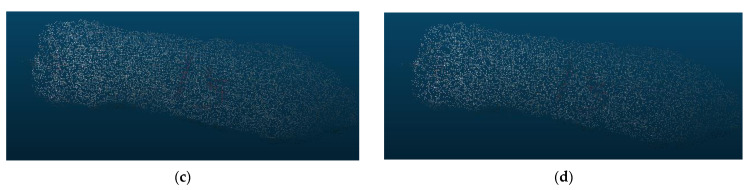
Down-sampling results of point clouds with varying voxel parameters. (**a**) Voxel parameter of 0.05; (**b**) voxel parameter of 0.06; (**c**) voxel parameter of 0.07; (**d**) voxel parameter of 0.08.

Selecting an appropriate down-sampling voxel parameter is critical to improving the efficiency of body dimension feature extraction. Our experiments have shown that as the parameter value for the voxel’s regular cube increases, the number of points retained after down-sampling decreases. This parameter value determines whether the down-sampled point cloud remains stable and representative. Figure 19 illustrates the down-sampling effects of different parameter values. When the voxel parameter is set to 0.005, the down-sampling rate is 85%. It ensures both the preservation of point cloud features and a significant reduction in point cloud segmentation computational load. More importantly, the computational efficiency gets enhanced.

### 3.4. Extraction Results of Point Cloud on the Back of Pig

In this research, the eigenvalues of envelope volume, envelope area, projection area, shoulder width, belly width, and hip width of the pig’s back were obtained. The partial extraction results of the pig’s back features are shown in Table 1. A Pearson coefficient test was employed to assess the back feature correlation of pigs. The correlation coefficients between different feature parameters are shown in Table 2. Among them, the correlation coefficients between shoulder width and envelope area and projection area are 0.753 and 0.759, respectively. This indicates that there is a substantial correlation between the characteristics.

### 3.5. Validation of Weight Estimation Model

To validate the accuracy of the pig weight estimation model, a comparison was delivered between the actual and estimated values of pig weights. Part of the results for the test dataset are presented in Table 3. It is evident from the table that the relative estimation error for the majority of pigs falls within 6%, which meets the production demands. Nevertheless, due to the relatively smooth surface of the weighing scale at the bottom of the data collection system, certain pigs may lean forward or backward, causing some features to be obscured or distorted. This can hinder the model’s ability to accurately extract the correct feature information, which would make it difficult to achieve precise weight estimation. It would be helpful to integrate a posture detection algorithm within the model for enhancing weight estimation accuracy.

### 3.6. Comparison of Different Weight Estimation Methods

**Figure 20:**
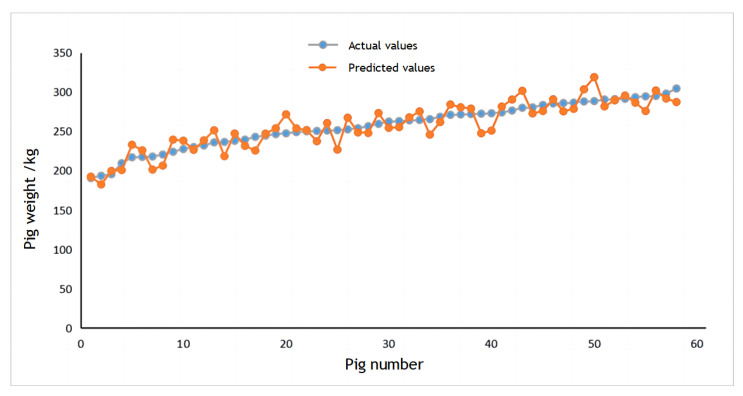
Scatter chart for weight estimation.

The dataset consisted of 140 pigs used as training samples for the CNN, with the remaining 58 pigs designated as test samples. The scatter plot between the true and estimated weight of pigs is shown in Figure 20. It demonstrates that the CNN-based pig weight estimation model exhibits a higher degree of accuracy in estimating pig weights.

**Figure 21:**
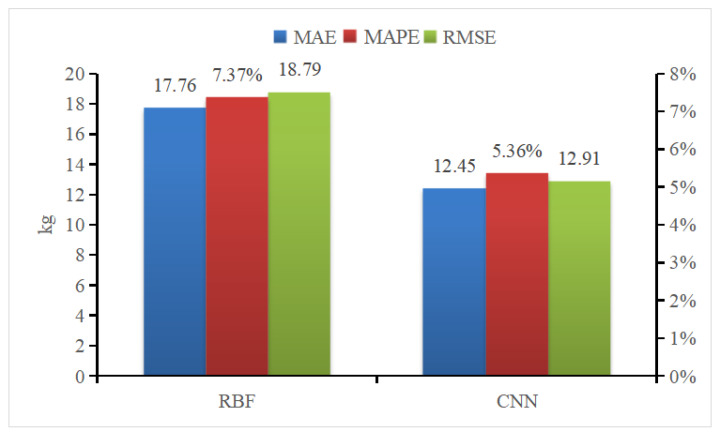
Comparison of pig weight estimation and evaluation indicators.

Radial Basis Function (RBF) Neural Networks was selected to compare the efficacy of different weight estimation model. RBF is a one-way propagation neural network with three or more layers that provides the most accurate approximation of nonlinear functions and the greatest global performance [39]. Figure 21 presents the results of the two weight estimation models using mean absolute error (MAE), mean absolute percent error (MAPE), and root mean square error (RMSE) as evaluation parameters. This comparison illustrates that all CNN parameters outperform those of RBF neural networks.

## 4. Discussion

### 4.1. Comparison with Previous Research

To evaluate the effectiveness and practicality of the algorithm proposed in this research, a comparative analysis is conducted in comparison to previous research. In reference [14], a 2D camera is employed to capture dorsal images of pigs, followed by the extraction of the dorsal image area of pigs. The correlation between dorsal area and weight is investigated, leading to the development of a model for estimating pig weight. In contrast, our proposed approach, building upon the extraction of dorsal projection area, extensively extracts various dorsal body dimension features, including envelope volume, envelope area, projection area, shoulder width, belly width, and hip width. All of these contribute to a more precise regression of pig weight. Leveraging a 3D-based weight estimation system allows for accurate capture of intricate details from various angles, effectively addressing distortions arising from changes in perspective and scale. This system provides enhanced accuracy and reliability in weight estimation. In the realm of 3D image-based weight estimation methodology, reference [20] employs four Kinect cameras to capture point cloud data of pigs, subsequently employing mesh reconstruction and deep neural network (DNN) to construct a weight estimation model. However, the utilization of multiple cameras, while comprehensive in capturing morphological features, presents challenges including high costs, intricate data fusion and processing, spatial constraints, and layout limitations, rendering it unsuitable for deployment in large-scale pig farms.

### 4.2. Deficiencies and Improvements

The postures of the pigs were found to influence the reliability and accuracy of back feature extraction. The change in pig postures impact the model’s feature extraction, resulting in deviations between the actual feature parameters and the extracted feature parameters, which will then affect the efficacy of the pig weight estimation model. In future research, we will consider the feature differences under various postures and construct different models to estimate the body weight of pigs in different postures.

**Figure 22:**
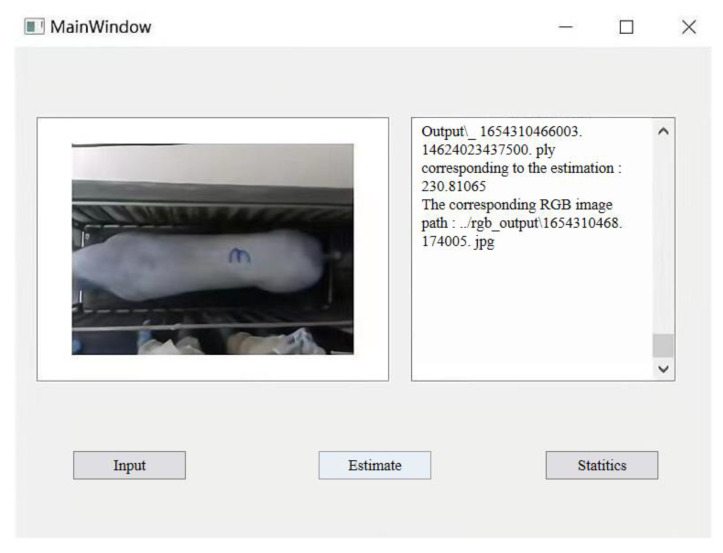
The weight management system.

Furthermore, due to uncontrolled movements of pigs, the acquired point cloud data are susceptible to noise, non-uniform or under-sampling, voids, and omissions, all of which can affect the effectiveness of body dimension parameter extraction. In future work, we plan to integrate multiple consecutive frames of point cloud data to densify and fill in the sparse and missing portions of the pig’s point cloud. To facilitate the practical implementation of non-contact weight estimation for pigs based on 3D imagery, further research will broaden the scope of target subjects and enhance the weight estimation model’s generalization capabilities. Drawing upon the output of pig weight estimation, we intend to assess the growth status of the pigs and offer tailored feeding recommendations. A weight management system was constructed in this research, as illustrated in Figure 22. The weight estimation model can be deployed on application terminals to provide information on pig weight gain status, weight-based feeding recommendations, and alert notifications.

## 5. Conclusions

This study introduces a 3D point cloud-based method for estimating pig weight, so as to address the challenges related pig weight monitoring and their susceptibility to stress. In this method, we separated the pig point cloud from the background using statistical filtering and DBSCAN, thereby resolving the problem of pig and background adhesion. Using convex hull detection, the point cloud data of the pig’s head and tail was removed in order to obtain complete point cloud data of their backs. Voxel down-sampling was used to reduce the number of point cloud data on the backs of pigs and enhance the weight estimation model’s efficiency and real-time performance. The weight estimation model based on back parameter characteristics and CNN was constructed. Also, the MAPE of the weight estimation model is only 5.357%, which could meet the demand of automatic weight monitoring in actual production.

It is worth mentioning that all the data used for training and validation in this method were collected from a real production environment, and only a depth camera above the drive channel was needed to achieve this method. Therefore, the method is easy to popularize and apply. What shall be noticed is that it can provide technical support for pig weight monitoring within both breeding and slaughterhouse settings.

## Figures and Tables

**Table 1:** Part of the pig characteristic parameter.

Number	Envelope Volume	Envelope Area	Projection Area	Shoulder Width	Belly Width	Hip Width
001	1.092	0.145	3.278	0.385	0.492	0.414
002	1.049	0.113	4.83	0.449	0.357	0.365
003	0.893	0.044	2.884	0.283	0.304	0.306
004	0.927	0.081	3.867	0.395	0.325	0.434
005	0.989	0.042	3.978	0.275	0.317	0.323
006	0.946	0.086	3.465	0.427	0.373	0.362
007	0.887	8.672	0.082	0.285	0.035	0.032
008	0.983	0.067	3.391	0.456	0.412	0.373
009	0.883	0.035	2.774	0.283	0.353	0.305
010	0.901	0.046	3.325	0.346	0.364	0.332

**Table 2:** Pearson correlation coefficient between different parameters.

	Envelope Volume	Envelope Area	Projection Area	Shoulder Width	Belly Width	Hip Width
**Envelope** **volume**	1	-	-	-	-	-
**Envelope** **area**	0.341	1	-	-	-	-
**Projection** **area**	0.264	0.686	1	-	-	-
**Shoulder** **width**	0.391	0.759	0.753	1	-	-
**Belly** **width**	0.223	0.442	0.547	0.661	1	-
**Hip** **width**	0.251	0.571	0.671	0.819	0.648	1

**Table 3:** Part of the results for the test dataset.

Pig No.	Actual Weight/kg	Estimated Weight/kg	Relative Error
1	238	246.2	3.865%
2	239.5	232.5	3.340%
3	243	226.6	7.160%
4	244.5	248.1	1.063%
5	246.5	252.8	2.961%
6	247.5	272.6	9.737%
7	249	252.8	1.927%
8	250	252.6	0.64%
9	250.5	236.4	5.229%
10	251	261.5	3.784%
11	251.5	225.7	9.860%
12	252.5	268.3	5.861%
13	254	247.6	2.125%
14	256.5	248.9	3.352%
15	259.5	274.2	5.279%
16	262.5	253.4	3.085%
17	263	256.3	2.927%
18	264	268.7	1.401%
19	264.5	274.3	4.083%
20	265.5	246.8	7.419%

## Data Availability

Not applicable.
